# Combined Effects of Ocean Warming and Acidification on Copepod Abundance, Body Size and Fatty Acid Content

**DOI:** 10.1371/journal.pone.0155952

**Published:** 2016-05-25

**Authors:** Jessica Garzke, Thomas Hansen, Stefanie M. H. Ismar, Ulrich Sommer

**Affiliations:** GEOMAR Helmholtz Centre for Ocean Research Kiel, Department of Marine Ecology, Experimental Ecology–Food Webs, Düsternbrooker Weg 20, 24105, Kiel, Germany; University of Western Sydney, AUSTRALIA

## Abstract

Concerns about increasing atmospheric CO_2_ concentrations and global warming have initiated studies on the consequences of multiple-stressor interactions on marine organisms and ecosystems. We present a fully-crossed factorial mesocosm study and assess how warming and acidification affect the abundance, body size, and fatty acid composition of copepods as a measure of nutritional quality. The experimental set-up allowed us to determine whether the effects of warming and acidification act additively, synergistically, or antagonistically on the abundance, body size, and fatty acid content of copepods, a major group of lower level consumers in marine food webs. Copepodite (developmental stages 1–5) and nauplii abundance were antagonistically affected by warming and acidification. Higher temperature decreased copepodite and nauplii abundance, while acidification partially compensated for the temperature effect. The abundance of adult copepods was negatively affected by warming. The prosome length of copepods was significantly reduced by warming, and the interaction of warming and CO_2_ antagonistically affected prosome length. Fatty acid composition was also significantly affected by warming. The content of saturated fatty acids increased, and the ratios of the polyunsaturated essential fatty acids docosahexaenoic- (DHA) and arachidonic acid (ARA) to total fatty acid content increased with higher temperatures. Additionally, here was a significant additive interaction effect of both parameters on arachidonic acid. Our results indicate that in a future ocean scenario, acidification might partially counteract some observed effects of increased temperature on zooplankton, while adding to others. These may be results of a fertilizing effect on phytoplankton as a copepod food source. In summary, copepod populations will be more strongly affected by warming rather than by acidifying oceans, but ocean acidification effects can modify some temperature impacts.

## Introduction

Anthropogenic activities have almost doubled the atmospheric carbon dioxide (CO_2_) concentration, and have driven both global warming and ocean acidification (OA) due to the greenhouse effect. The uptake of CO_2_ by the surface ocean has caused profound changes in marine carbonate chemistry: increased aqueous CO_2_, bicarbonate (HCO_3_^-^), and hydrogen ion (H^+^) concentrations, while the concentration of carbonate ions (CO_3_^2-^) declined [1]. Contemporary surface ocean pH has declined by 0.1 units since pre-industrial time [2]. Simultaneously ocean sea surface temperature is predicted to increase up to 3–5°C by the year 2100 [3]. The consequences of ocean warming and OA for planktonic organisms remain unclear as only a few studies to-date have experimentally tested their combined effects on natural plankton communities [4–8]. Particularly the effects on plankton phenology (directly modifying food quantity) and physiology (modifying food quality) remain unresolved.

Studies show diverse effects of ocean acidification on corals [9], molluscs [10], crustaceans [11], echinoderms [12], and fish [13]. Responses of marine organisms to acidification range from reduced skeletogenesis, increased mortality, and altered growth, to changes in development and abundance [14–17]. Responses of marine organisms vary significantly between species and location. Whereas growth of calcifying taxa is typically negatively affected by acidification, growth in diatoms and fleshy algae can be enhanced, and species shifts may result [15].

It is known, that warming seawater alter phenology (earlier peak occurrences) [18], species distribution (biogeographical species shifts)[19], community composition (shift to smaller species) [20], lower densities [21], and reduced body sizes [20,22]. Higher temperature can affect individuals with subsequent changes to entire ecosystems, through increasing metabolic rates [23], higher energy demands [24], increased consumption rates [25], as well as accelerated development and growth, which consequently alters food-web structures and can have major implications for higher trophic levels [25].

An increasing number of studies suggest that the combined effects of temperature and *p*CO_2_ prevail on diverse marine (e.g. [14,15]) and freshwater taxa (e.g. [26]) by affecting their survival, calcification, growth and abundance [14,15,27,28]. Interacting stressors can affect organisms in three ways: (1) through additive effects (sum of the individual effects), (2) synergistic effects (combined effects are greater than the sum of the individual effect), and (3) antagonistic effects (stressor offsets the effect of the other)[15]. Meta-analyses highlight that responses to more than one stressor are trait-, taxa-, life stage- and habitat specific, reflecting local adaptations. Studies that address effects of OA and warming on marine organisms, especially primary producers, show that these factors can lead to reductions in quality in terms of macromolecular composition [7,11,29] and consequently impact the nutritional value for higher trophic levels that depend upon these primary producers as a source of essential biomolecules to gain energy for growth, reproduction and development. [11,30,31]. OA and warming effects on marine organisms are found to alter energy budgets due to reduced performance curves under interacting stressors [32].

Metabolic energy, in form of lipids (hereafter called fatty acids (FA)) is one important metabolic trait that can adapt to changing environmental conditions (i.e. nutrient availability temperature and CO_2_ concentration). FAs consist of hydrocarbon chains of different length and grade of saturation (identified by number of double bounds). Generally, FAs are classified into saturated (SFA, no double bonds), monounsaturated (MUFA, one double bond), and polyunsaturated fatty acids (PUFA, with two or more double bonds). Among these fatty acids, some PUFAs are essential and cannot be synthesised de novo and have to be taken up via diet. PUFAs are documented to be crucial for copepod egg production, hatching, growth and development [33]. Also higher trophic levels, fish and their larvae, depend on the fatty acid composition in their food for successful recruitment and reproduction (e.g. [34]). The fatty acid composition in marine micro-algae, the major food source of copepods, differs between taxonomic groups and therefore fatty acids can be used as trophic markes, e.g 22:6(n-3) (DHA; docosahexaenoic) and 20:5(n-3) (EPA; eicosapentaenoic acid) for flagellates and diatoms respectively.

Calanoid copepods constitute approximately 80% of the global zooplankton and are the dominant trophic link between phytoplankton and fish [35]. It is known that warming decreases body size, survival and reproductive success [22], studies have shown the opposite for other organisms [11]. Additionally, warming leads to changes in fatty acid composition [35], but to-date it remains unclear how ocean acidification will affect fatty acid composition [11]. It is unknown whether the effects of warming and acidification mutually enhance or reciprocally dampen the responses of marine copepods. Short-term studies have shown no effects on eggs, larvae and adults at up to 2000 μatm pCO2 [36–40].

We conducted a mesocosm experiment in the Kiel Fjord, Western Baltic Sea, an area where plankton is already exposed to strong short-term and seasonal variations in *p*CO_2_ (summer and autumn variation 375–2300 μatm; summer and autumn means: 700 μatm [41]). Therefore, we expected direct detrimental effects of CO_2_ on copepods, as well as indirect food web effects; and interactive effects with rising temperatures could not be excluded *a priori*, and need to be tested. Populations from highly pH fluctuating ecosystems might be adapted and therefore less sensitive than other populations of the same species of different ecosystem locations [10]. Based on previous single stressor studies of warming [22] and acidification effects [42], we hypothesized that warming and acidification would mainly lead to antagonistic responses in copepod abundance, body size and fatty acid composition.

## Materials and Methods

### Ethic statement

No specific permission was required for activities related to field sampling. The field location was not privately owned or protected, and neither endangered nor protected species were involved.

### Experimental design

**Fig 1:**
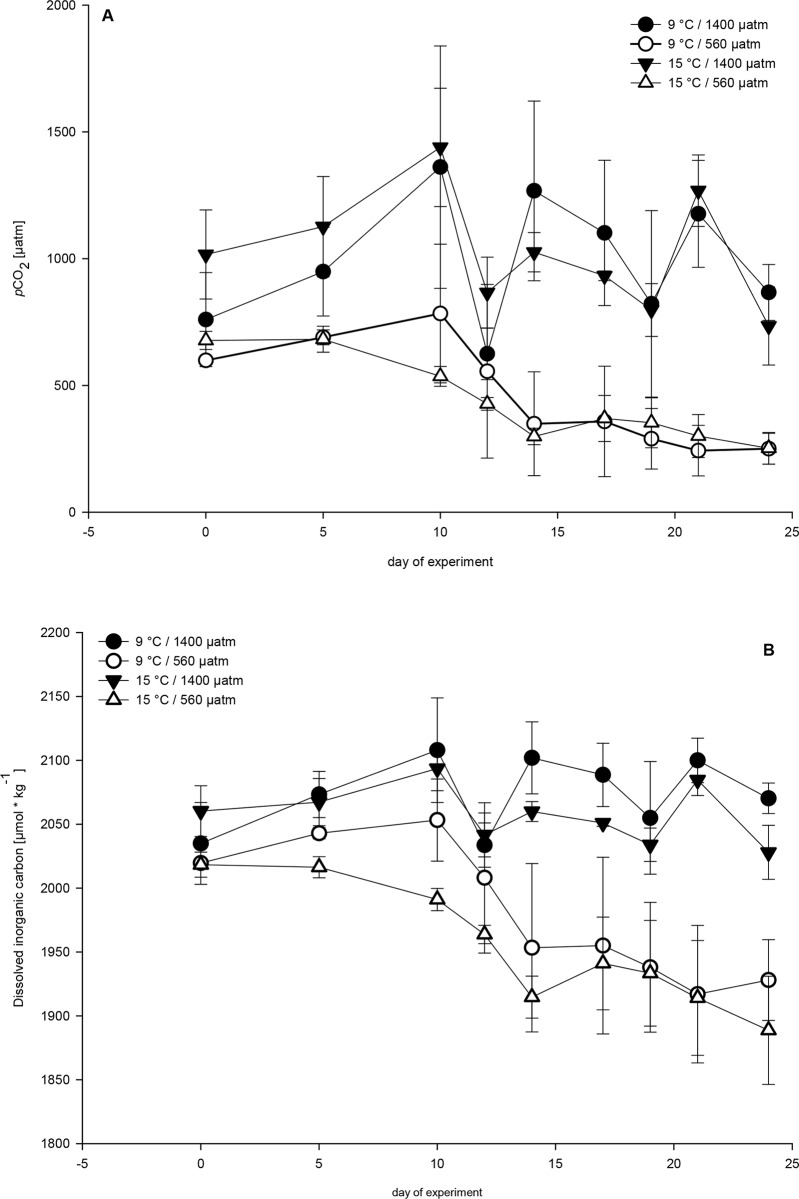
**Temporal development** of (A) mean *p*CO_2_ and (B) mean dissolved inorganic carbon of each treatment. Error bars denote ± 1 SE (n = 3). Open symbols represent high *p*CO_2_ (1400 μatm) and closes symbols low *p*CO_2_ (560 μatm) concentrations. Symbols for the treatment combinations as in key.

A 24-day mesocosm experiment was conducted October 19th to November 12th 2012. Unfiltered seawater from Kiel Fjord (54° 20′ N, 10° 8′ E) was used to fill twelve indoor mesocosms, each with a volume of 1400 L. Mesocosms contained the natural early autumn plankton community composition of algae, bacteria and protozoa. To minimize between-mesocosm differences in the initial community compositions and densities of phytoplankton, seawater was pumped from approximately 2 m depth into a mixing chamber by a rotary pump. From this mixing chamber the water was simultaneously filled into the mesocosms. Mesozooplankton from net catches (Kiel Bight) were added at target concentrations of 20 individuals L^-1^, to mimic natural densities of copepods during this season. The plankton was gently stirred by a propeller to homogeneously mix the water column without incurring mesozooplankton mortality. A full-factorial replicated experiment (n = 3 per treatment) was used with two temperature regimes (9°C and 15°C, ∆3°C of ambient temperature) and two *p*CO_2_ levels (560 μatm, hereafter called “low”, and 1400 μatm *p*CO_2_, hereafter called “high”). The target levels mimic the extent of warming and acidification predicted for this season (October 19th to November 12th 2012) and region of the IPCC prediction (Scenario IS92a, atmospheric CO_2_: 788μatm) for the year 2100, when the surface seawater CO2 in the Baltic Sea is predicted to reach 1400μatm and higher [41,43]. The resulting set-up of twelve mesocosms was installed in four temperature-controlled culture rooms. Target temperatures and levels of *p*CO_2_ were reached in all treatments three days after filling (19 October 2012), which will henceforth be called day -3 (Fig 1 and S1 Fig). Temperature deviation in a mesocosm between day 0 and day 21 was a maximum of ± 0.3°C. Maximal temperature deviation between mesocosm in the same temperature treatment was 0.3°C (for the warm) and 0.4°C (for the cold treatment).

CO_2_ manipulation was monitored during the experimental period using a flow of 30–60 L h^-1^ CO_2_-enriched air (low: 560 μatm; high: 1400 μatm CO_2_) through the headspace of the mesocosms. To balance the natural draw-down of CO_2_ by phytoplankton production that occurred during the course of the experiment, CO_2_ enriched water was added to the high CO_2_ mesocosms three times (October 29th, November 2nd and 9th) [44]. For this purpose, water taken from the mesocosms was filtered (0.2 μm pore size), CO_2_-saturated by bubbling, and again transferred into the mesocosms. The required volumes were calculated on the basis of dissolved inorganic carbon (DIC) and total alkalinity (Fig 1 and S1 Fig).

Over the course of the experiment, light supply and day length were adjusted according to the seasonal patterns expected at this latitude and season. Light was supplied by computer controlled light units (GHL Groß Hard- und Softwarelösungen, Lampunit HL3700 and ProfiluxII). Above each of the mesocosms, one unit consisted of 5 HIBay-LED spotlights (purpose-built item of Econlux, each 100 W) was installed. Daily irradiance patterns were set to follow the pattern for a cloudless 21st September at Kiel (according to Brock [45]) and reduced to 50% to account for moderate under water light attenuation. The light-dark cycle was 11h50 min: 12h10 min. The daily maximum light intensity in the middle of the water column was 252 μmol m^-2^ s^-1^ PAR.

### Sampling and measurements

Total dissolved inorganic carbon (DIC) samples (10 mL) were taken on each sampling day with glass vials (Resteck, Germany) filled using a peristaltic pump (flow rate 6 mL min^-1^) and an intake tube containing a single syringe filter (0.2 μm, Sartorius). Filtered samples were fixed with saturated HgCl_2_ solution (20 μL), crimped with a headspace of less than 1% and stored in the dark at 4°C. DIC was measured following Hansen et al. [46] using a SRI-8610C (Torrence, USA) gas chromatograph. For total alkalinity (TA), 25 mL samples were filtered (Whatman GF/F filter 0.2 μm) and titrated at 20°C with 0.05M HCl-solution in an automated titration device (Metrohm Swiss mode). The remaining carbonate parameter *p*CO_2_ was calculated using CO2SYS [47] and the constants supplied by Hansson [48] and Mehrbach et al. [49], that were refitted by Dickson and Millero [50].

Water temperature, salinity and pH were measured daily (S1 Fig). Phytoplankton was sampled three times per week (Monday, Wednesday, and Friday), and fixed with Lugol’s iodine, and subsequently identified to species level. To estimate phytoplankton biomass, density and cell size were measured and converted to carbon biomass following Hillebrand et al. [51]. Zooplankton was sampled weekly by three vertical net hauls, with a hand-held plankton net (64 μm mesh size, 12 cm diameter, and from 150 cm depth) and fixed with Lugol’s iodine. Each net haul sampled a volume of approximately 5.1 L. The total zooplankton catch was divided in a sample splitter (HydroBios, Germany), so that ¼ of the total catch volume was counted and identified, and copepod developmental stages and sexes could be distinguished accurately [22]. The body length constancy between moults enables a clear assignment of size to a given stage. All copepods were identified to genus level by using a ZEISS Discovery V.8 microscope with the magnification between 25x and 40x [22].

Body size of taxonomically identified copepods was measured digitally via photographs and digital software (ZEISS AxioVision 4.8 and AxioCam MRc) with a precision to the nearest μm [22]. Means were calculated stage-specifically for copepods of each genus found in each mesocosm (see S1 Table). The mean developmental index was calculated following Ismar et al. [52]:

$$DI=\sum \left(NS\right)/N_{tot}$$

Where N = number of copepods at certain stage, S = assigned stage value, N_tot_ = total number of copepods staged. Stages were scored as: C1 = 1, C2 = 2, C3 = 3, C4 = 4, C5 = 5, adult = 6.

To analyze copepod total fatty acid content and fatty acid composition, 30 adult *Paracalanus* sp. individuals were pooled in tin cups and extracted in chloroform / dichlormethane / methanol (1:1:1 v/v/v) following Arndt and Sommer [53]. Prior to extraction two internal standards, heneicosanoic acid (C21) and FAME mix (C19) were added. Methyl esters were prepared by esterification with toluene and H_2_SO_4_ (1%) in methanol heated up to 50°C for 12 hours. After extraction with n-hexane, the fatty acid methyl esters were analyzed with a gas chromatograph (Thermo Scientific Trace GC Ultra with autosampler AS 3000), comparing peaks against the standards FAME Mix C4-C24 SUPELCO, Sigma-Aldrich, Germany.

The observed combined impact of both stressors was compared with their expected net additive effect [(*stressor*1_*warm/low*_ – *control*_*cold/low*_)+(*stressor*2_*cold/high*_ – *control*_*cold/low*_)], which was based on the sum of their individual effects. If the observed combined response of both stressors exceeded their expected additive response then interaction was identified as being synergistic. In contrast, if the observed response was less than the additive response, the interaction was denoted as antagonism [26]. For illustration of interaction response types, we show graphically the difference between observed [(*stressor*_*warm/high*_ – *control*_*cold/low*_)] and predicted additive effects, which indicates the direction and magnitude of the interaction.

### Statistical analysis

We tested for the combined effects of temperature and *p*CO_2_ on the: (1) abundance of copepodits (stage C1-C5), adults, and nauplii of all occurring taxa, (2) prosome length of adult copepods, (3) stage-specific prosome length of *Paracalanus* sp., (4) mean developmental index, and (5) fatty acid ratios. Normal distribution of all response variables was tested by using Kolmogorov-Smirnov analyses for temperature and *p*CO_2_ effects on all response variables (α = 0.05), data were normally distributed and no transformation was needed.

Temperature and *p*CO_2_ levels were set as categorical explanatory variables, in separate, 2-way ANOVAs with abundance, mean developmental index, and fatty acid ratios as respective continuous response variables. Phytoplankton biomass was analysed by cross-correlation through time within mesocosms and followed by ANOVA with cross-correlation coefficients for temperature and *p*CO2 effects. 3-way ANOVAs were used to identify prosome length differences in response to temperature, *p*CO_2_ and both stressors in respect to species (differences in adult prosome length) and developmental stages (differences in *Paracalanus* sp.). Tukey’s honest significant difference test was used as the post hoc test for all ANOVAs. To test homogeneity of variance, Fligner-Killeen tests were applied in all cases. All statistical tests were conducted at a significance threshold of α = 0.05. All statistical analyses were conducted in R, Version 0.97.551, R Inc. using the packages stats, multcomp, and car.

## Results

### Abundance

**Fig 2:**
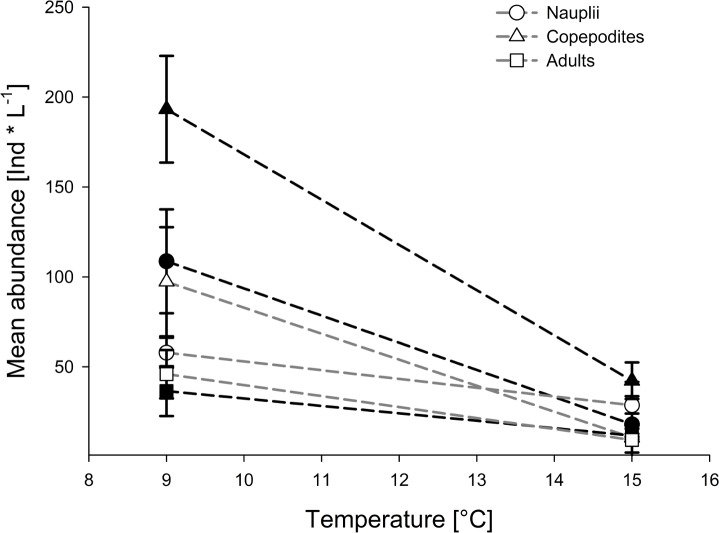
Mean abundance of nauplii, copepodites and adults of the last experimental day. Error bars denote ± 1 SE (n = 3). Open symbols represent high *p*CO_2_ (1400 μatm) and closes symbols low *p*CO_2_ (560 μatm) concentrations. Symbols for the treatment combinations as in key.

**Fig 3:**
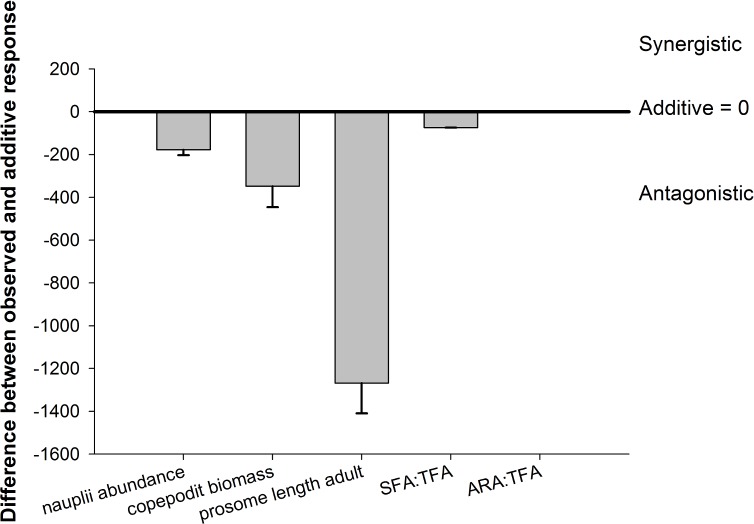
The nature (synergistic, additive, antagonistic) and magnitude of the observed interactions of temperature and OA on copepods. Error bars denote ± 1 SE (n = 3).

Temperature and *p*CO_2_ interacted significantly and affected nauplii abundance antagonistically with the lowest abundance under high temperature/high *p*CO_2_ (Table 1, Figs 2 and 3). OA and warming decreased nauplii abundance by 30.90% compared to the low temperature/low *p*CO_2_ treatment (S2 Table), although the nauplii abundance was lowest under OA and warming (Fig 2). Warming decreased the abundance significantly by 27.96% (Table 1 and S2 Table). Overall, nauplii abundance responses to OA differed significantly between the two temperature treatments, and OA thus had no significant first-order effect on the abundance of nauplii (Tables 1 and 2).

**Table 1:** Results of 2-way ANOVA explaining the effects of temperature (temp.) and *p*CO_2_ on abundance of nauplii, copepodites (C1-C5), adults, and mean developmental index (MDI). Values in bold are significant at p < 0.05.

Variable	Factor	df	Sum Sq.	Mean Sq	F	p-value
**Nauplii**	Temp	1	10784	10784	320. 70	**<0.01**
	*p*CO_2_	1	1216	1216	3.68	0.09
	Temp x *p*CO_2_	1	2830	2830	8.58	**<0.05**
	Residuals	8	2638	330		
**Copepodites**	Temp	1	42372	42372		**<0.01**
	*p*CO2	1	12220	12220		**<0.01**
	Temp x *p*CO_2_	1	3089	3089		**<0.05**
	Residuals	8	3805	476		
**Adults**	Temp	1	2821.3	2821.3	18.99	**<0.01**
	*p*CO_2_	1	36.1	36.1	0.24	0.64
	Temp x *p*CO_2_	1	106.4	106.4	0.72	0.42
	Residuals	8	1188.5	148.6		
**MDI**	Temp	1	5.08	5.08	7.45	**<0.05**
	*p*CO_2_	1	0.99	0.99	1.47	0.26
	Temp x *p*CO_2_	1	1.38	1.38	2.02	0.19
	Residuals	8	5.46	0.68		

**Table 2:** Tukey Honest Significance Test results on abundance of nauplii, copepodites (C1-C5), adults, and the mean developmental index (MDI). Values in bold are significant at p <0.05.

*Variable*	*9°C vs. 1°C*	*560 μatm vs. 1400 μatm*
**Nauplii**	**<0.001**	0.09
**Copepodites**	**<0.001**	**<0.001**
**Adults**	**<0.01**	0.64
**MDI**	**<0.05**	0.26

Warming and OA had a significant antagonistic effect on the abundance of copepodites (Table 1 and Fig 3), with an increase of 10.25% compared to the low temperature-low *p*CO_2_ treatment (S2 Table). Warming alone significantly decreased abundance of copepodites by 27.47% within the low *p*CO_2_ treatment; whereas acidification significantly increased copepodite abundance by 38.10% compared to both low temperature treatments (Figs 2 and 3, Tables 1 and 2, S2 Table).

Abundance of adult copepods was significantly negatively affected by warming but neither by *p*CO_2_ nor by the interaction of both stressors (Tables 1 and 2). Warming decreased adult copepod abundance by 67.64% compared to the low temperature treatment (S2 Table).

**Fig 4:**
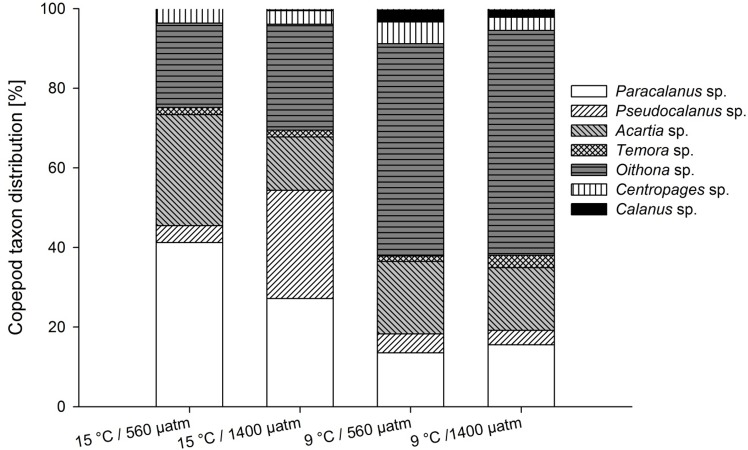
Mean copepod taxon distribution for all treatments of the last experimental day. Symbols for the taxon as in key.

The copepod community included the genera *Paracalanus*, *Pseudocalanus*, *Oithona*, *Acartia*, *Temora*, and *Calanus*. *Oithona* sp. dominated in cold treatments, while *Paracalanus* sp. in warm treatments (Fig 4). The mean developmental index (MDI) was significantly affected by temperature with the highest MDI in high temperature/high *p*CO2 treatments and the lowest under low temperature/high *p*CO2 conditions (Tables 1 and 2). At higher temperatures, the MDI indicated a dominance of the copepodite stages C2 and C3, whereas copepods at lower temperature had on average to between copepodite stages C1 and C2, indicating that copepodites developed faster in warmer treatments (Table 3). The differences in MDI indicated a phenological shift between temperature treatments. The MDI showed neither a response to *p*CO2 nor to the interaction of both stressors (Tables 1 and 2).

**Table 3:** Mean developmental indices (MDI) (± 1SD) (see S1 Table to find individual stage abundances).

	MDI (± 1 SD)
**9°C / 560 μatm**	1.87 (±0.09)
**9°C / 1400 μatm**	1.77 (±0.21)
**15°C / 560 μatm**	2.49 (±0.58)
**15°C / 1400 μatm**	2.87 (±0.24)

The cross-correlation coefficients for warming and acidification impacts on edible phytoplankton biomass as a copepod food source were negative at the beginning of the experiment, and positive during the mid-phase and the end of the experiment (S3 Table and S2 Fig). The ANOVA analysis of all correlation coefficients over the experimental time in all tanks indicated that food biomass was significantly affected by *p*CO_2_ (S4A and S4B Table). Higher food biomass was found under high *p*CO_2_ and was neither affected by temperature nor by the interaction of both stressors. Consequently, high *p*CO_2_ had a significantly positively affected copepod biomass (S4A and S4B Table).

### Body size

**Fig 5:**
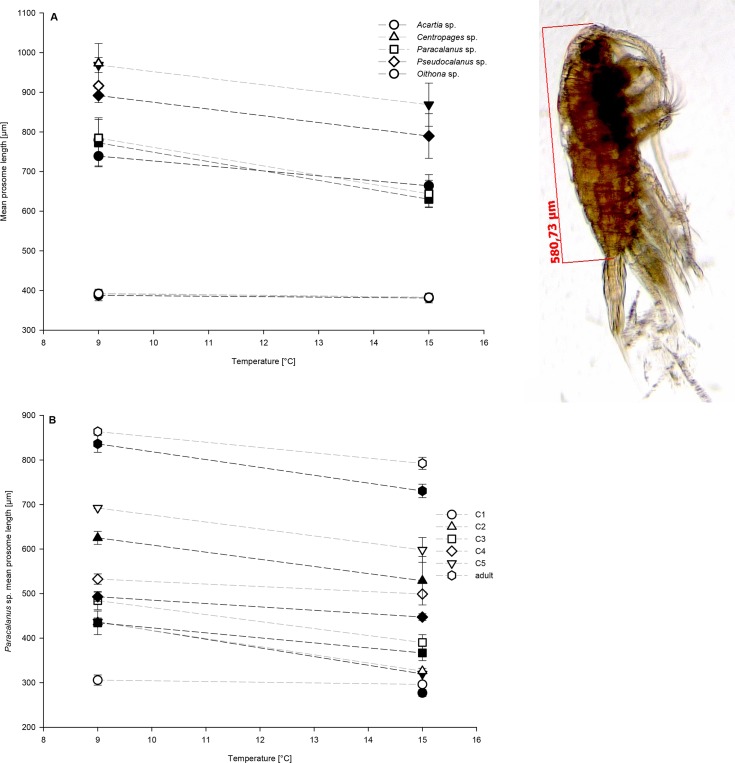
**Mean prosome lengths in μm of the last experimental day** of (A) all occurring adults of each taxon and (B) all developmental stages of *Paracalanu*s sp. Open symbols represent high *p*CO_2_ (1400 μatm) and closes symbols low *p*CO_2_ (560 μatm) concentrations. Symbols for the treatment combinations as in key. Error bars denote ± 1 SE (n = 3).

The mean prosome length of adult copepods of all occurring species was significantly affected by the interaction of temperature and *p*CO_2_, as well as temperature and *p*CO_2_ alone (Tables 4 and 5, Fig 5A). The interaction of OA and warming antagonistically affected adult prosome length of all species (Fig 3), with the smallest prosome lengths at high temperature/low *p*CO_2_ compared to low temperature/high *p*CO_2_ conditions (Fig 5A). Overall high temperature decreased prosome lengths of adults and high *p*CO_2_ increased mean prosome lengths, irrespective of the copepod species (Tables 4 and 5, Fig 5A), resulting in an antagonistic interaction of warming and OA on adult copepod prosome length (Fig 3).

**Table 4:** ANOVA results explaining the effects of temperature (temp) and *p*CO_2_ on prosome lengths of adults (all occurring species) and *Paracalanus* sp. (all developmental stages). Values in bold are significant at p <0.05.

*Variable*	*Factor*	*df*	*F*	*p-value*
Adults	Temp	1	34.59	**<0.01**
	Temp x *p*CO_2_	1	5.59	**<0.05**
	Temp x species	3	2.65	0.08
	*p*CO_2_ x species	4	4.68	**<0.01**
	Temp x *p*CO_2_ x species	1	0.27	0.61
	Residuals	18		
*Paracalanus* sp.	Temp	1	24.34	**<0.01**
	*p*CO_2_	1	0.01	0.93
	Temp x *p*CO_2_	1	0.72	0.40
	Temp x *p*CO_2_ x stage	1	0.11	0.74
	Residuals	32		

**Table 5:** Tukey Honest Significance Test results on prosome length of adults and of all developmental stages of *Paracalanus* sp. Values in bold are significant at p <0.05.

*Variable*	*9°C vs. 15°C*	*560 μatm vs. 1400 μatm*
Adults	**<0.001**	**<0.01**
*Paracalanus* sp.	**<0.001**	0.93

*Paracalanus* sp. was abundant enough for an analysis across most developmental stages. Prosome size changes with temperature and OA were specifically analysed for stage-specific changes (Fig 5B). Warming only significantly affected mean stage-specific prosome lengths; neither OA nor the interaction of both factors affected the mean prosome lengths significantly (Tables 4 and 5, Fig 5B). Overall, warming decreased the mean prosome lengths in all developmental stages compared to low temperature (C1: -1.33%; C2: -26.86%; C3: -14.53%; C4: -8.94%; C5: +0.03%; adults: -18.47%)(S2 Table). Even if OA did not affect significantly mean prosome lengths of *Paracalanus* stages; prosome lengths increased with higher *p*CO_2_ compared to the low *p*CO_2_ treatments (see S2 Table). On average, adult *Paracalanus* sp. were 21.29 μm larger at lower temperature compared to high temperature, and 9.82 μm smaller at low *p*CO_2_ compared to high *p*CO_2_ treatments.

### Fatty acid composition

**Fig 6:**
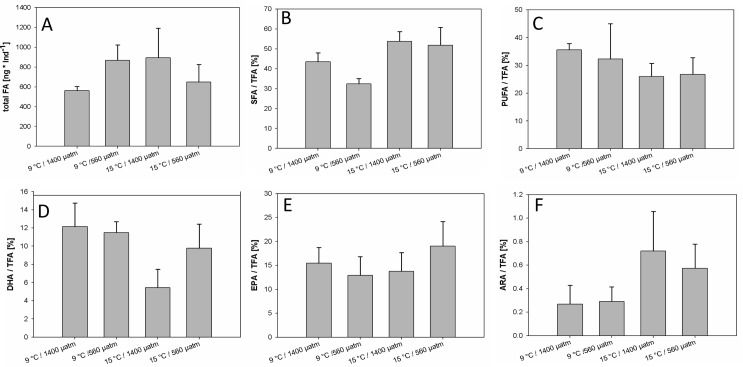
Mean fatty acid composition of one adult *Paracalanus* sp. of the last experimental day. (A) total fatty acid (TFA) content, (B) saturated FA / TFA, (C) polyunsaturated FA / TFA, (D) DHA / TFA, (E) EPA / TFA, (F) ARA / TFA (raw FA data S5 Table). Error bars denote for ± 1 SD (n = 3).

The total amount of fatty acids per adult individual of *Paracalanus* sp. was not significantly influenced by temperature, OA or their interaction (Tables 6 and 7, Fig 6A). The ratio of saturated fatty acids-to-total fatty acids (SFA/TFA) was significantly affected by the interaction of temperature and OA, with the lowest amount of SFA/TFA under low temperature/low *p*CO_2_ and the highest amount at higher temperature/high *p*CO_2_ (Fig 6B), resulting in an antagonistic interaction effect (Fig 3). SFA/TFA ratio increased by 45.25% under OA and warming compared to low temperature/low *p*CO_2_, but the differences between both OA treatments at higher temperatures were not significant (Table 7 and Fig 6B, S2 Table). Warming increased SFA/TFA by 47.90% and OA by 17.90% compared to low temperature and *p*CO_2_ treatments, respectively, and therefore combined factors of OA and warming affected SFA/TFA antagonistically (Fig 3, S2 Table); the temperature effect dominated the response of SFA/TFA ratio more strongly than the effect of high *p*CO_2_.

**Table 6:** Results of 2-way ANOVA explaining the effects of temperature (temp) and *p*CO_2_ on fatty acids. Values in bold are significant at p < 0.05 (S5 Table to find single fatty acid quantities).

*Variable*	*Factor*	*dF*	*F*	*p-value*
**SFA:TFA**	Temp	1	25.37	**<0.01**
	*p*CO_2_	1	0.18	0.68
	Temp x *p*CO_2_	1	7.53	**<0.05**
	Residuals	7		
**PUFA:TFA**	Temp	1	2.38	0.17
	*p*CO_2_	1	0.66	0.44
	Temp x *p*CO_2_	1	0.01	0.92
	Residuals	7		
**DHA:TFA**	Temp	1	5.78	**<0.05**
	*p*CO_2_	1	0.06	0.82
	Temp x *p*CO_2_	1	0.31	0.59
	Residuals	7		
**EPA:TFA**	Temp	1	0.69	0.43
	*p*CO_2_	1	0.24	0.64
	Temp x *p*CO_2_	1	0.10	0.76
	Residuals	7		
**ARA:TFA**	Temp	1	19.35	**<0.01**
	*p*CO_2_	1	7.29	**<0.05**
	Temp x *p*CO_2_	1	7.06	**<0.05**
	Residuals	7		

**Table 7:** Tukey Honest Significance Test results on fatty acid ratios of female adult Paracalanus sp. Values in bold are significant at p <0.05.

*Variable*	*9°C vs. 15°C*	*560 μatm vs. 1400 μatm*
**SFA:TFA**	**<0.01**	0.68
**PUFA:TFA**	0.17	0.45
**DHA:TFA**	**<0.05**	0.72
**EPA:TFA**	0.58	0.95
**ARA:TFA**	**<0.01**	0.10

The ratio of polyunsaturated-to-TFA content (PUFA/TFA) was neither significantly affected by temperature, *p*CO_2_ nor their interaction (Tables 6 and 7). But even without significant effects of all treatments, lower PUFA/TFA ratios were measured at higher temperature treatments compared to ambient temperature treatments (Fig 6C). Overall, warming decreased PUFA/TFA ratios by 24.89% (S2 Table). The responses in polyunsaturated essential fatty acids, which cannot be synthesised *de novo* by heterotrophic organisms like copepods, were analysed for temperature, and OA effects, and their interactions. The DHA/TFA ratio (docosahexaenoic acid (22:6(n-3)) was significantly affected by temperature, but neither by OA, or the interaction of both (Tables 6 and 7). The lowest proportion of DHA/TFA was found in the high temperature and low *p*CO_2_ treatment (Fig 6D) and warming decreased the DHA/TFA by 35.73% (S2 Table).

The ratio of EPA/TFA (eicosapentaenoic acid (20:5(n-3)) was neither affected by temperature, *p*CO_2_ or their interaction (Tables 6 and 7, Fig 6E). ARA/TFA ratios (arachidonic acid (20:4(n-4))) were significantly affected by the interaction of temperature and OA, and by both single factors, respectively (Tables 6 and 7). The lowest ARA/TFA ratio was found under high temperature and low *p*CO_2_ conditions, and the highest ratio under high temperature and high *p*CO_2_ (Fig 6F); warming and OA additively affected ARA/TFA ratio (Fig 3), and their interaction significantly increased the ratio by 167.57% (S2 Table).

## Discussion

Our results indicate that in a future ocean scenario, temperature effects will dominate zooplankton responses, with acidification partially counteracting some observed effects of increased temperature, whilst adding to others. The abundance of adult copepods in our study was negatively affected by warming, while OA impacts could modify temperature effects on the abundance of younger life stages. Warming significantly reduced the prosome length of copepods and the interaction of warming CO_2_ antagonistically affected prosome length, but not significantly. Fatty acid composition was also significantly affected by warming. The content of saturated fatty acids increased, and the ratios of the polyunsaturated essential fatty acids docosahexaenoic- (DHA), and arachidonic acid (ARA) to total fatty acid content increased with higher temperatures. Additionally, arachidonic acid content significantly increased with higher *p*CO_2_, and there was a significant additive interaction effect of both parameters. In summary, copepod populations will likely be affected more strongly by warming than by acidifying oceans, but ocean acidification effects can modify temperature impacts on zooplankton. Our results indicate that body size will be impacted by OA and temperature antagonistically, while showing additive OA interactions with rising temperatures on aspects zooplankton nutritional composition. Each of these responses is discussed in more detail below.

### Abundance

The observed interactive effect of warming and OA on nauplii abundance suggests that the reproductive success of copepods during the experiment was sensitive to higher temperatures and higher *p*CO_2_; nauplii were more abundant in treatments that were exposed to only higher temperature or only higher *p*CO_2_. Studies observed that nauplii stages seem to be more vulnerable to environmental changes than others (i.e. CO_2_ concentration [54,55], invertebrates [27,56], and other marine organisms [27,28]. Several factors can lead to the lower number of nauplii under warming and OA: 1) females might have produced a lower number of eggs [40,55], 2) hatching success may have been reduced due to a higher number of unfertilized eggs, non-viable fecund eggs or viable fecund eggs in a quiescent state [57], or 3) higher mortality rates [58].

Copepodite stages (C1-C5) were also significantly affected by the interaction of warming and OA, but the antagonistic effect was lower than within the nauplii. Especially OA positively affected copepodites, whereas warming decreased the copepodite abundance. Younger developmental stages seem to be more vulnerable to OA than older ones, especially adults seem to be highest tolerant to OA effects as our study has shown [59,60]. Temperature seemed to be the more important driver within older developmental stages of marine copepods. Higher temperatures alone affected the stage composition and shifted the MDI from a copepodite stage mixture of 1 and 2 to dominance in copepodite stage mixture of 2 and 3 in warmer treatments; no effect of CO_2_ was detected. This can be explained by faster maturation of copepods at higher temperatures. At higher temperatures, copepods developed faster to the reproductive stage and can reproduce earlier [23,61]. Higher daily mortality and lower stage-specific survival rate [22] at higher temperatures may have resulted in the lower abundance of copepods observed in our samples. A positive effect of OA on Baltic Sea copepods was experimentally identified by Rossoll et al. [42], they concluded that this is most likely the response to a higher food biomass. Thus the findings of Rossoll et al. [42] provide an experimental explanation for our results of higher copepod abundances at high *p*CO_2_ treatments, where phytoplankton biomass was also higher [6]. During this mesocosm experiment temperature mostly negative affected phytoplankton biomass during the bloom, Paul et al. [7] observed a 50% decline in bloom phytoplankton biomass and an increase of carbon-to-phosphorus ratio by 5–8%. The stoichiometry (C:P) of food resources are important for copepod developmental rates and less balanced nutrient ratios can lead to slower development, growth or increased respiration rates [62,63]. Increased energetic costs to persist environmental changes lead to altered metabolic allocation to accommodate increased energetic expenses. Imbalances in metabolism can decrease lifetime fitness du to re-allocation of energy [32,64,65]. Yet, food limitation can be excluded as a sole direct causal factor for the observed changes in mesozooplankton abundance. Edible phytoplankton biomass was not significantly correlated with copepod biomass. The significant temperature-acidification interaction term suggested that acidification partially reversed the negative influence of warming on abundance. These antagonistic effects on zooplankton abundance were also observed in a limnic multi-stressor study of Christensen et al. [26]. Calanoid copepods like *Acartia* sp. might be able to compensate higher metabolic costs at higher temperature by increased consumption rates and higher food availability in acidification treatments. Additionally, copepods originating from the Kiel Fjord seem to be acid-tolerant due to pre-adaptation in the high *p*CO_2_ fluctuation environment [43]. Christensen et al. [26] argued that the limnic species *Daphnia catawba*, an acid-tolerant herbivore, benefits more from acidification than acid-sensitive competitors due to the positive effects of warming on feeding rates and growth.

### Body size

This study shows that warming and OA interactively affected adult copepod body size; warming decreased and higher *p*CO_2_ concentration increased adult body sizes. These antagonistic results suggest that the smaller adult body size at higher temperatures can be partially compensated by the positive effect of OA. Indirect positive CO_2_ effects on copepod size via the effect on food availability are indicated from our cross-correlation results. Phytoplankton biomass data, used as a proxy for available food biomass in our experiment, demonstrated that acidification acted as a fertilizer for phytoplankton but that higher temperature treatments had lower phytoplankton biomass compared to the low temperature/low *p*CO_2_, which was also experimentally shown in other studies [11,66]. The results of a negative effect of higher temperature are in line with results of a meta-analysis of Daufresne et al. [20] and an experimental study of Garzke et al. [22]. The Temperature-Size Rule (TSR) [61] describes the growth response of ectothermic organisms to temperature by which individual organisms grow faster at higher temperatures, but attain smaller sizes at maturity than at lower temperatures. Size at a defined stage is a product of growth rate (increase in biomass per time) and developmental rate (increase in life stage per time, and the TSR signals that both rates are decoupled [67]. Forster et al. [68] showed that the developmental rate in marine pelagic copepods has a greater temperature dependence across all life stages than growth rates. We could show that the relative size change increased with higher life history stage resulting in greater difference of adult sizes between the climate change scenarios (warming, OA, and warming x OA) compared to the low temperature/low *p*CO_2_. Body size changes with OA in copepods were not investigated directly within on species between the different developmental stages. Only a few studies have investigated the effects of OA or the interaction of warming and OA on body mass. Hildebrand et al. [69] experimentally showed that body mass (here body carbon) decreased with increasing *p*CO_2_ as well as a decrease of dry weight of the arctic copepod species *Calanus hyperboreus*. Hildebrand et al. [69] argued that the decrease in body carbon resulted due higher energetic costs under OA for the acid-base regulation. Our results suggest that ocean acidification may have weaker effects on copepod body size compared to ocean warming. Havenhand [70] suggested that the ecologically most important groups of the Baltic Sea food web (phytoplankton, zooplankton, macrozoobenthos, cod, and sprat) seem to be more or less well adapted to future acidification but more vulnerable to higher temperatures

### Fatty acid composition

This study presents first species-specific experimental results of combined global change effects, ocean acidification and warming, on the fatty acid composition of a natural community of marine pelagic copepods. Such a study is particularly important for understanding the observed phenological changes (abundance and body size) by the impact of environmental changes to biochemical components that are most important for reproduction, development and growth. Only one study that we are aware of analysed the fatty acid composition of copepods of a natural community [71]. Mayor et al. [71] analyses the community fatty acid composition using metabolomics and stated that predicted future scenarios of OA and warming unlikely significantly impair copepod metabolism. Bermudez et al. [29] were the first to study the effects of OA and warming on the diatom *Cylindrotheca fussiformes* and found that the interaction of both stressor affect the total PUFA composition. Interestingly, they also found no significant OA effect on PUFAs in the warming treatment, whereas at lower temperature the PUFA amount decreased with higher *p*CO_2_ [29]. During this study fatty acid composition of adult *Paracalanus* sp. was generally stronger affected by temperature rather by *p*CO_2_. SFA-to-TFA ratios increased with warming, as did the essential FAs, ARA/TFA, whereas only DHA/TFA ratios decreased with warming. EPA/TFA was neither affected by warming nor by OA. The temperature effects on SFA-to-TFA ratios and high *p*CO_2_ are consistent with observations of Rossoll et al. [11]. The increase of the SFA proportion can be explained by more SFA incorporated into the cell membranes to maintain viscosity at higher temperatures and to counteract the decreasing internal cell-pH with acidification [72]. Further, SFA are also classified as fast fuel energy and SFA production might be up-regulated under warming and OA to counteract the increased energetic costs to persist in warmer and acidified environments [71].

Like other animals, copepods have nutritional requirements of essential ω-3 and ω-6 fatty acids, which must be supplied by their food resources. DHA, EPA and ARA are important essential FAs for promoting growth, development and reproduction [33,73] as well as for egg production and hatching success of copepods [74,75]1. Our experimental results showed a lower amount of DHA per TFA at higher temperature, which might have caused the lower abundance of nauplii because Evjemo et al. [75] and Rossoll et al. [11] could experimentally show that a decreased DHA amount reduces the egg production a female adults. We did not measure daily egg production, but nauplii abundances on the last experimental day were significantly lower at higher temperature and at low *p*CO_2_. These nauplii had hatched during the last days of the experiment in the different treatments and reflect treatment-dependent egg production [76], daily survival [22], and developmental rates [11]. The additive effect of warming and acidification on ARA/TFA proportion might have led to the abundance changes in nauplii. Data from fish showed that higher concentrations of the n-6 FA family reduces the egg production and hatching success [77]. The reproductive rate of copepods can be negatively affected by ARA excess especially when the inadequate composition of essential FAs lead to metabolic disorder [75,78]. If the relative essential FA composition of the diet, e.g. DHA/ARA proportion, is not optimal for the animal, this might lead to disfunctionality or reduced health/fitness [75]

We suggest that enhanced developmental rates at higher temperatures increase the need of essential FA for building reproductive tissue and egg production. If food sources have lower amounts of these essential PUFAs, reproductive success is reduced. Since our study did not include fatty acid and stoichiometry analyses of phytoplankton, we cannot determine the specific change in algal profile. However, since the measured PUFAs constitute essential fatty acids, these effects in mesozooplankton were necessarily food-derived, and it is known from single species studies that algae under high *p*CO_2_ and higher temperatures are a lower quality food source for copepods [11,63].

We suggest that temperature increases, within the regional IPCC predictions for the year 2300, will mainly decrease individual body size, copepod abundance and change FA composition of copepods in the Baltic Sea. Further, we suggest that acidification effects, within the IPCC predictions, may have a positive effect on copepod size and abundance by promoting phytoplankton growth as their main food source, and higher food biomass availability, which may to some extent ameliorate the temperature-induced higher energy expense. In fact, studies have found a positive effect of CO_2_ on phytoplankton community growth or biomass when mesograzers were excluded prior to the experiments [79]. Yet, negative effects of increasing CO_2_-levels may synergistically with warming impede the nutritional quality of copepods for fish. Warming and acidification combined might lead to food limitation of copepods, which are less able to cover their enhanced metabolism.

Our results highlight the prevalence and magnitude of interacting anthropogenic stressors. Several studies have demonstrated the complex impacts of multiple stressors on several species. The results showed a diverse picture of sensitivities and the nature of responses among different taxa and ecosystems [6,7,9,12,28,29]. Przeslawski et al. [28] summarized in their meta-analysis that marine early life stages are more of synergistically affected by multi-stressor interactions (65%) than additively (17%) or antagonistically (17%). But all these studies also mention that populations can be locally adapted to different environmental conditions and respond differently to the same acidification stress [15,41]. The Kiel Fjord strongly fluctuates in *p*CO_2_ concentration during the year so that organisms might be tolerant for high *p*CO_2_ concentrations [41,43]. Animals that regularly face exposure to variable seawater temperature and *p*CO_2_ might be more resilient due to physiological adaptation [80]. Here, we tested the responses of a community, which in contrast to other single-species studies showed antagonistic effects for copepods. Studies with multi-species assemblages showed opposite impacts of multiple stressor scenarios due to compensatory dynamics among tolerant species [14,15,42].

Our findings have implications for higher trophic levels in marine food webs. Smaller copepod prosome lengths and lower abundances at higher temperatures could reduce the matter and energy transfer through the food web, affecting higher trophic levels such as fish larvae, which themselves have increased metabolic rate demands of energy [23]. Changes of the fatty acid composition, especially within the essential PUFAs, may have consequences for fish development. The observed ratio changes of the essential FAs DHA and ARA to the total FA content with warming and acidification has the potential to affect the successful rearing of marine fish, for which the fatty-acid ratio and the amount play an important role.

From our results we predict that food web impacts of simultaneously increasing pCO2 and ocean temperature on higher trophic levels may vary under different scenarios: they may depend on whether higher trophic level consumers are food quality- or quantity limited, which needs to be tested case-specifically.

## Supporting Information

S1 Fig **Temporal development of (A) mean total alkalinity, (B) mean dissolved inorganic carbon DIC), and (C) temperature of each treatment.** Error bars denote ± 1 SE (n = 3). Open symbols represent high *p*CO_2_ (1400 μatm) and closes symbols low *p*CO_2_ (560 μatm) concentrations. Symbols for the treatment combinations as in key.
(TIFF)

S2 Fig (A) Temporal mean biomass of edible phytoplankton [μg C * L^**-1**^] and (B) mean copepod abundance (C1-adult) [ind * L^**-1**^**].** Error bars denote for ± 1 SD (n = 3). Open symbols represent high *p*CO_2_ (1400 μatm) and closes symbols low *p*CO_2_ (560 μatm) concentrations. Symbols for the treatment combinations as in key.
(TIFF)

S1 Table Species- and stage specifically raw data abundances [individuals L^-1^]. (DOCX)

S2 Table Magnitudes of change between treatments compared to the 9°C / 560 μatm treatment. OA (9°C / 1400 μatm), OW (ocean warming: 15°C / 560 μatm), and OW/OA (15°C / 1400 μatm). Values in bold notice significant ANOVA results (see Tables 1A, 4A and 5A) at p < 0.05.
(DOCX)

S3 Table Cross-correlation coefficients of phytoplankton and zooplankton biomass (see S2 Fig). (DOCX)

S4 Table **(A) ANOVA results of correlation coefficients of phytoplankton *vs* zooplankton biomass. (B) Tukey Honest Significance Test results on phytoplankton *vs* zooplankton biomass correlation coefficients.** Values in bold are significant at p <0.05.
(DOCX)

S5 Table Fatty acid content (ng per individual) of adult female *Paracalanus* sp. of the last experimental day. (DOCX)
