# Supplementary figures and images for: Combined Effects of Ocean Warming and Acidification on Copepod Abundance, Body Size and Fatty Acid Content
Source: PLoS One. 2016 May 25;11(5):e0155952. doi: 10.1371/journal.pone.0155952 (PMC4880321; doi:10.1371/journal.pone.0155952)

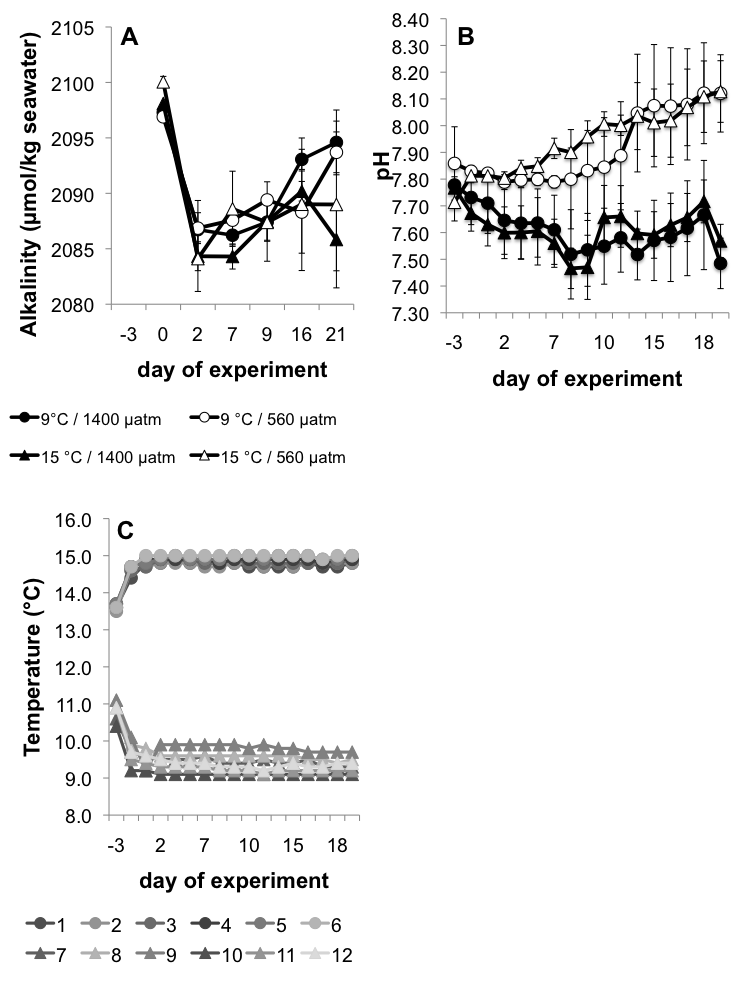

Supplement: S1 Fig — Temporal development of (A) mean total alkalinity, (B) mean dissolved inorganic carbon DIC), and (C) temperature of each treatment. Error bars denote ± 1 SE (n = 3). Open symbols represent high pCO2 (1400 μatm) and closes symbols low pCO2 (560 μatm) concentrations. Symbols for the treatment combinations as in key. (TIFF) [file pone.0155952.s001.tiff]

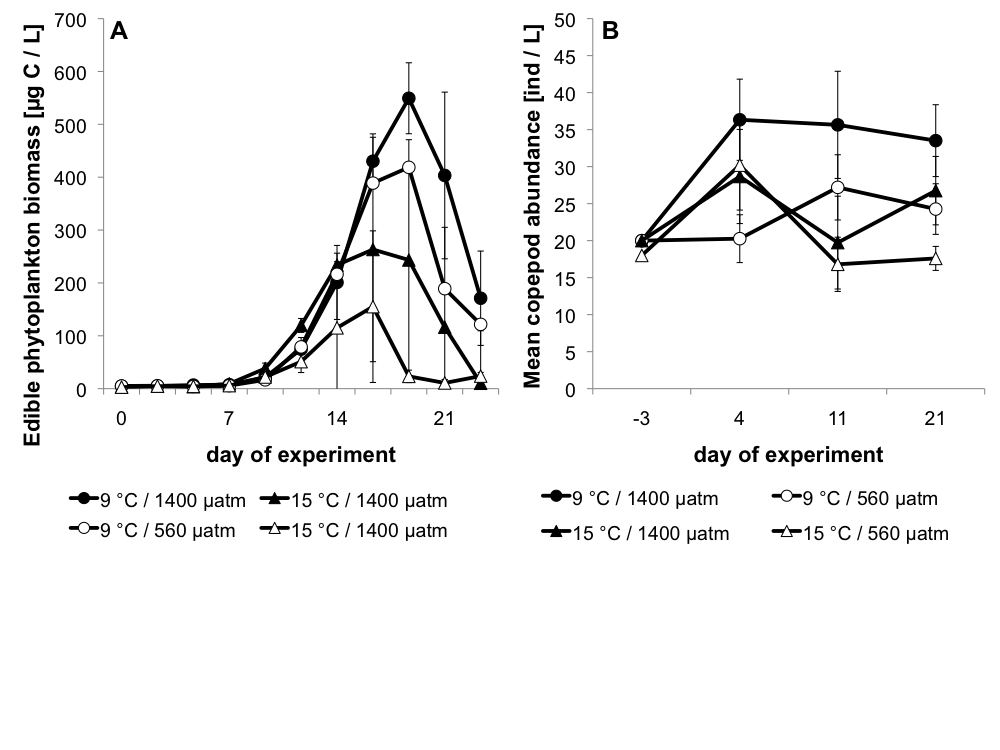

Supplement: S2 Fig — (A) Temporal mean biomass of edible phytoplankton [μg C * L-1] and (B) mean copepod abundance (C1-adult) [ind * L-1]. Error bars denote for ± 1 SD (n = 3). Open symbols represent high pCO2 (1400 μatm) and closes symbols low pCO2 (560 μatm) concentrations. Symbols for the treatment combinations as in key. (TIFF) [file pone.0155952.s002.tiff]
