# Supplementary material for: Combined Effects of Ocean Warming and Acidification on Copepod Abundance, Body Size and Fatty Acid Content
Source: PLoS One. 2016 May 25;11(5):e0155952. doi: 10.1371/journal.pone.0155952 (PMC4880321; doi:10.1371/journal.pone.0155952)
Supplement: S1 Table — (DOCX) [file pone.0155952.s003.docx]

S1Table. Species- and stage specifically raw data abundances (individuals per liter).

| species | Stage value | 15 °C / 560 µatm | 15 °C / 560 µatm | 15 °C / 560 µatm | 15 °C / 1400 µatm | 15 °C / 1400 µatm | 15 °C / 1400 µatm | 9 °C / 560 µatm | 9 °C / 560 µatm | 9 °C / 560 µatm | 9 °C / 1400 µatm | 9 °C / 1400 µatm | 9 °C / 1400 µatm |
| --- | --- | --- | --- | --- | --- | --- | --- | --- | --- | --- | --- | --- | --- |
| Nauplii | 1 | 13.60 | 34.93 | 37.33 | 26.13 | 28.00 | 0.00 | 53.87 | 52.27 | 67.47 | 106.4 | 138.67 | 81.07 |
| *Pseudo-/ Paracalanus* | 2 | 0.80 | 0.53 | 2.133 | 2.93 | 2.13 | 0.53 | 1.33 | 1.60 | 0.80 | 2.93 | 2.13 | 2.67 |
| *Pseudo-/ Paracalanus* | 3 | 1.60 | 0.80 | 6.400 | 3.47 | 4.80 | 0.533 | 1.07 | 2.13 | 3.20 | 5.87 | 7.73 | 1.60 |
| *Pseudo-/ Paracalanus* | 4 | 3.47 | 3.47 | 9.600 | 7.73 | 1.87 | 5.60 | 0.00 | 2.40 | 0.80 | 0.53 | 0.27 | 0.80 |
| *Paracalanus* sp. | 5 | 2.67 | 1.87 | 8.53 | 5.07 | 3.20 | 5.07 | 0.267 | 0.800 | 0.27 | 0.27 | 0.80 | 0.80 |
| *Paracalanus* sp. | 6 | 1.60 | 0.00 | 1.33 | 4.80 | 2.93 | 3.20 | 0.800 | 0.27 | 0.00 | 0.27 | 0.00 | 0.00 |
| *Paracalanus* sp. | adult | 0.00 | 0.000 | 2.13 | 0.27 | 1.07 | 0.27 | 2.40 | 0.27 | 0.27 | 1.73 | 3.20 | 1.60 |
| *Pseudocalanus* sp. | 5 | 0.27 | 0.27 | 0.27 | 0.00 | 0.00 | 0.27 | 0.00 | 0.27 | 0.00 | 0.27 | 0.00 | 0.53 |
| *Pseudocalanus* sp. | 6 | 0.27 | 0.00 | 0.27 | 0.00 | 0.80 | 0.53 | 0.53 | 0.267 | 0.00 | 0.00 | 0.00 | 0.27 |
| *Pseudocalanus* sp. | adult | 0.27 | 0.00 | 0.27 | 0.00 | 0.00 | 0.00 | 0.53 | 0.27 | 0.00 | 0.13 | 0.27 | 0.53 |
| *Acartia* sp. | 2 | 0.00 | 0.27 | 2.13 | 1.07 | 0.53 | 0.00 | 0.80 | 0.00 | 0.80 | 0.40 | 0.53 | 2.93 |
| *Acartia* sp. | 3 | 0.00 | 0.27 | 1.60 | 0.27 | 0.53 | 1.33 | 2.13 | 0.27 | 1.87 | 0.13 | 1.07 | 1.60 |
| *Acartia* sp. | 4 | 0.00 | 0.27 | 2.40 | 0.00 | 1.07 | 1.33 | 0.27 | 0.00 | 0.27 | 0.00 | 0.27 | 0.27 |
| *Acartia* sp. | 5 | 0.00 | 0.00 | 3.47 | 0.00 | 1.87 | 2.67 | 0.27 | 0.00 | 0.00 | 0.40 | 0.53 | 0.27 |
| *Acartia* sp. | 6 | 0.27 | 0.00 | 1.60 | 0.00 | 0.27 | 1.87 | 0.00 | 0.27 | 0.00 | 0.00 | 0.00 | 0.00 |
| *Acartia* sp. | adult | 0.00 | 0.00 | 0.00 | 0.00 | 0.00 | 0.00 | 0.00 | 0.00 | 0.27 | 0.13 | 0.00 | 0.27 |
| *Temora* sp. | 2 | 0.00 | 0.00 | 0.27 | 0.00 | 0.00 | 0.27 | 0.00 | 0.00 | 0.00 | 0.00 | 0.00 | 0.27 |
| *Temora* sp. | 3 | 0.00 | 0.00 | 0.27 | 0.00 | 0.53 | 0.00 | 0.00 | 0.53 | 0.00 | 0.00 | 0.00 | 0.27 |
| *Temora* sp. | 4 | 0.00 | 0.00 | 0.00 | 0.00 | 0.00 | 0.00 | 0.00 | 0.00 | 0.00 | 0.13 | 0.00 | 1.07 |
| *Temora* sp. | 5 | 0.00 | 0.00 | 0.00 | 0.00 | 0.53 | 0.00 | 0.00 | 0.00 | 0.00 | 0.00 | 0.00 | 0.00 |
| *Temora* sp. | 6 | 0.00 | 0.00 | 0.00 | 0.00 | 0.27 | 0.00 | 0.00 | 0.00 | 0.00 | 0.00 | 0.00 | 0.00 |
| *Temora* sp. | adult | 0.27 | 0.00 | 0.00 | 0.00 | 0.00 | 0.00 | 0.00 | 0.00 | 0.00 | 0.00 | 0.00 | 0.00 |
| *Oithona* sp. | 2 | 0.00 | 0.00 | 0.00 | 0.00 | 0.00 | 0.00 | 0.00 | 0.00 | 0.00 | 0.00 | 0.27 | 0.00 |
| *Oithona* sp. | 3 | 0.00 | 0.00 | 0.27 | 0.27 | 0.27 | 0.00 | 0.00 | 0.27 | 0.00 | 0.00 | 0.00 | 0.00 |
| *Oithona* sp. | 4 | 0.00 | 0.00 | 0.00 | 0.53 | 0.27 | 0.00 | 0.27 | 0.27 | 0.27 | 0.00 | 0.00 | 0.00 |
| *Oithona* sp. | 5 | 0.00 | 0.00 | 2.13 | 0.00 | 1.60 | 0.27 | 1.87 | 2.13 | 2.93 | 0.93 | 0.80 | 1.87 |
| *Oithona* sp. | 6 | 0.00 | 0.80 | 0.53 | 0.27 | 1.60 | 1.07 | 2.13 | 1.60 | 2.67 | 1.87 | 0.80 | 5.33 |
| *Oithona* sp. | adult | 0.53 | 1.87 | 3.20 | 3.20 | 2.40 | 13.60 | 0.00 | 3.73 | 2.93 | 4.13 | 10.3 | 4.53 |
| *Centropages* sp. | 2 | 0.27 | 0.00 | 0.53 | 0.00 | 0.00 | 0.27 | 0.27 | 0.53 | 0.00 | 0.00 | 0.0 | 0.27 |
| *Centropages* sp. | 3 | 0.00 | 0.00 | 0.00 | 0.00 | 0.27 | 0.00 | 0.27 | 0.27 | 0.00 | 0.00 | 0.00 | 0.27 |
| *Centropages* sp. | 4 | 0.00 | 0.00 | 0.00 | 0.00 | 0.27 | 0.27 | 0.00 | 0.00 | 0.00 | 0.00 | 0.00 | 0.53 |
| *Centropages* sp. | 5 | 0.00 | 0.00 | 0.53 | 0.00 | 0.80 | 0.80 | 0.00 | 0.00 | 0.00 | 0.00 | 0.00 | 0.27 |
| *Centropages* sp. | 6 | 0.00 | 0.00 | 0.00 | 0.27 | 0.00 | 0.27 | 0.00 | 0.00 | 0.27 | 0.00 | 0.00 | 0.00 |
| *Centropages* sp. | adult | 0.00 | 0.00 | 0.27 | 0.00 | 0.00 | 0.00 | 0.00 | 0.00 | 0.53 | 0.00 | 0.00 | 0.53 |
| *Calanus* sp. | 2 | 0.00 | 0.00 | 0.00 | 0.00 | 0.00 | 0.00 | 0.00 | 0.00 | 0.00 | 0.00 | 0.00 | 0.27 |
| *Calanus* sp. | 3 | 0.00 | 0.00 | 0.00 | 0.00 | 0.00 | 0.00 | 0.00 | 0.00 | 0.00 | 0.00 | 0.00 | 0.00 |
| *Calanus* sp. | 4 | 0.00 | 0.00 | 0.00 | 0.00 | 0.00 | 0.00 | 0.00 | 0.00 | 0.00 | 0.00 | 0.00 | 0.00 |
| *Calanus* sp. | 5 | 0.00 | 0.00 | 0.00 | 0.27 | 0.00 | 0.27 | 0.27 | 0.53 | 0.27 | 0.13 | 0.00 | 0.27 |
| *Calanus* sp. | 6 | 0.00 | 0.00 | 0.00 | 0.00 | 0.00 | 0.00 | 0.27 | 0.00 | 0.00 | 0.00 | 0.53 | 0.00 |
| *Calanus* sp. | adult | 0.00 | 0.00 | 0.00 | 0.00 | 0.00 | 0.00 | 0.00 | 0.00 | 0.00 | 0.00 | 0.00 | 0.00 |
