# Supplementary material for: Combined Effects of Ocean Warming and Acidification on Copepod Abundance, Body Size and Fatty Acid Content
Source: PLoS One. 2016 May 25;11(5):e0155952. doi: 10.1371/journal.pone.0155952 (PMC4880321; doi:10.1371/journal.pone.0155952)
Supplement: S2 Table — OA (9°C / 1400 μatm), OW (ocean warming: 15°C / 560 μatm), and OW/OA (15°C / 1400 μatm). Values in bold notice significant ANOVA results (see Tables 1A, 4A and 5A) at p < 0.05. (DOCX) [file pone.0155952.s004.docx]

**S2 Table:**

|  | ***OA*** | ***OW*** | ***OA / OW*** |
| --- | --- | --- | --- |
| **Abundance** | |  |  |
| Nauplii | +79.79 % | **-27.96%** | **-30.90%** |
| Copepods | **+38.10%** | **-27.47%** | **+10.25%** |
| Adult | +25.79% | **-67.64%** | -74.45% |
|  |  |  |  |
| ***Paracalanus* sp. prosome length** | |  |  |
| Adult | +1.54% | **-18.47%** | -16.71% |
| C5 | +10.58% | **+0.03%** | +1.73% |
| C4 | +7.95% | **-8.94%** | +0.21% |
| C3 | +11.29% | **-14.53%** | -10.45% |
| C2 | +0.25% | **-26.86%** | -25.20% |
| C1 | +1.99% | **-1.33%** | -7.57% |
|  |  |  |  |
| **Fatty Acids** |  |  |  |
| SFA/TFA | +17.90% | **+47.90%** | **+45**.25% |
| PUFA/TFA | +3.46% | -24.89% | -25.61% |
| DHA/TFA | 16.72% | **-35.73%** | -31.57% |
| EPA/TFA | -13.33% | -26.60% | -14.85% |
| ARA/TFA | **-16.07%** | **+13.96%** | **+167.57%** |
|  |  |  |  |
