# Supplementary material for: Combined Effects of Ocean Warming and Acidification on Copepod Abundance, Body Size and Fatty Acid Content
Source: PLoS One. 2016 May 25;11(5):e0155952. doi: 10.1371/journal.pone.0155952 (PMC4880321; doi:10.1371/journal.pone.0155952)
Supplement: S3 Table — (DOCX) [file pone.0155952.s005.docx]

**Table S3:**

| *Experimental day* | *treatment* | *CI* |
| --- | --- | --- |
| 4 | 9 °C / 560 µatm | 0.71 |
| 4 | 9 °C / 1400 µatm | -0.45 |
| 4 | 15 °C / 560 µatm | -0.20 |
| 4 | 15 °C / 1400 µatm | -0.48 |
| 11 | 9 °C / 560 µatm | 0.66 |
| 11 | 9 °C / 1400 µatm | 0.21 |
| 11 | 15 °C / 560 µatm | 0.93 |
| 11 | 15 °C / 1400 µatm | -0.34 |
| 21 | 9 °C / 560 µatm | 0.89 |
| 21 | 9 °C / 1400 µatm | -0.23 |
| 21 | 15 °C / 560 µatm | 0.50 |
| 21 | 15 °C / 1400 µatm | 0.28 |
