# Supplementary material for: Combined Effects of Ocean Warming and Acidification on Copepod Abundance, Body Size and Fatty Acid Content
Source: PLoS One. 2016 May 25;11(5):e0155952. doi: 10.1371/journal.pone.0155952 (PMC4880321; doi:10.1371/journal.pone.0155952)
Supplement: S4 Table — (A) ANOVA results of correlation coefficients of phytoplankton vs zooplankton biomass. (B) Tukey Honest Significance Test results on phytoplankton vs zooplankton biomass correlation coefficients. Values in bold are significant at p <0.05. (DOCX) [file pone.0155952.s006.docx]

**Table S4:**

| A |  |  |  |  |  |
| --- | --- | --- | --- | --- | --- |
| Factor | **df** | **Sum Sq.** | **Mean Sq** | **F** | **p-value** |
| Temperature | 1 | 0.10 | 0.10 | 0.57 | 0.49 |
| *p*CO_2_ | 1 | 1.69 | 1.69 | 9.49 | **<0.05** |
| day | 1 | 0.38 | 0.38 | 2.13 | 0.22 |
| Temperature x *p*CO_2_ | 1 | 0.08 | 0.08 | 0.43 | 0.55 |
| Temperature x day | 1 | 0.14 | 0.14 | 0.76 | 0.43 |
| *p*CO_2_ x day | 1 | 0.01 | 0.01 | 0.01 | 0.91 |
| Temperature x *p*CO_2_ x day | 1 | 0.01 | 0.01 | 0.03 | 0.86 |
| Residuals | 8 | 0.71 | 0.18 |  |  |
| **B** |  |  |  |  |  |
| **9 °C vs. 15 °C** |  | **560 vs. 1400 µatm** | | |  |
| 0.44 |  | **<0.05** |  |  |  |
