# Supplementary material for: Combined Effects of Ocean Warming and Acidification on Copepod Abundance, Body Size and Fatty Acid Content
Source: PLoS One. 2016 May 25;11(5):e0155952. doi: 10.1371/journal.pone.0155952 (PMC4880321; doi:10.1371/journal.pone.0155952)
Supplement: S5 Table — (DOCX) [file pone.0155952.s007.docx]

S2Table. Fatty acid content (ng per individual) of adult female *Paracalanu*s sp. of the last experimental day.

| treatment | 15 °C/1400 µatm | 9 °C/560 µatm | 15 °C/1400 µatm | 9 °C/560 µatm | 9 °C/560 µatm | 9 °C/560 µatm | 15 °C/560 µatm | 15 °C/560 µatm | 9 °C/1400 µatm | 9 °C/1400 µatm | 15 °C/1400 µatm | 15 °C/1400 µatm | 9 °C/560 µatm | 15 °C/1400 µatm | 9 °C/560 µatm |
| --- | --- | --- | --- | --- | --- | --- | --- | --- | --- | --- | --- | --- | --- | --- | --- |
| Fatty Acid |  |  |  |  |  |  |  |  |  |  |  |  |  |  |  |
| 08:00 | 0.000 | 0.000 | 0.000 | 0.000 | 0.000 | 0.000 | 0.000 | 0.000 | 0.000 | 0.000 | 0.000 | 0.000 | 0.000 | 0.000 | 0.000 |
| 10:00 | 0.000 | 0.000 | 0.000 | 0.000 | 0.372 | 0.000 | 0.362 | 0.362 | 0.000 | 0.105 | 0.000 | 0.000 | 0.000 | 0.000 | 0.000 |
| 11:00 | 0.000 | 0.000 | 0.000 | 0.000 | 0.000 | 0.000 | 0.000 | 0.000 | 0.000 | 0.000 | 0.000 | 0.000 | 0.000 | 0.000 | 0.000 |
| 12:00 | 0.000 | 0.000 | 2.887 | 2.810 | 1.998 | 0.904 | 1.934 | 1.934 | 1.280 | 1.243 | 0.949 | 0.908 | 2.450 | 2.596 | 2.431 |
| 13:00 | 0.000 | 0.000 | 1.177 | 0.942 | 0.000 | 0.271 | 0.630 | 0.630 | 0.320 | 0.357 | 0.000 | 0.126 | 0.000 | 0.935 | 0.000 |
| 14:00 | 47.307 | 18.809 | 78.883 | 62.284 | 41.813 | 24.828 | 60.534 | 60.534 | 45.424 | 20.995 | 42.778 | 56.815 | 41.691 | 42.756 | 59.969 |
| 14:01 | 0.268 | 0.275 | 0.842 | 0.742 | 5.865 | 2.631 | 0.522 | 0.522 | 6.481 | 3.133 | 1.450 | 0.692 | 6.113 | 1.363 | 0.597 |
| 00:00 | 2.999 | 2.762 | 8.335 | 4.900 | 4.482 | 2.658 | 5.141 | 5.141 | 3.839 | 3.899 | 3.373 | 2.218 | 4.657 | 4.029 | 5.705 |
| 15:01 | 0.629 | 0.997 | 2.673 | 2.821 | 1.249 | 0.133 | 0.796 | 0.796 | 0.197 | 0.290 | 0.250 | 0.951 | 2.741 | 0.867 | 2.160 |
| 16:00 | 132.418 | 99.252 | 345.700 | 163.219 | 185.644 | 115.299 | 212.685 | 212.685 | 118.234 | 118.658 | 132.123 | 116.576 | 237.514 | 248.571 | 255.607 |
| 16:01 | 4.401 | 81.393 | 167.841 | 115.368 | 91.599 | 49.759 | 92.561 | 92.561 | 80.842 | 35.006 | 55.155 | 71.340 | 86.370 | 69.297 | 182.958 |
| 17:00 | 13.728 | 11.836 | 23.093 | 19.854 | 14.996 | 7.415 | 17.616 | 17.616 | 12.856 | 6.386 | 12.488 | 15.277 | 12.053 | 8.993 | 28.259 |
| 17:01 | 12.026 | 14.201 | 10.422 | 13.538 | 20.210 | 5.753 | 12.062 | 12.012 | 4.201 | 3.954 | 16.136 | 21.397 | 9.071 | 3.471 | 18.371 |
| 18:00 | 63.711 | 48.982 | 204.559 | 59.578 | 64.640 | 80.669 | 139.653 | 139.653 | 44.339 | 71.730 | 76.494 | 57.959 | 125.583 | 180.089 | 123.727 |
| 18:1n9t | 0.000 | 0.000 | 0.186 | 0.000 | 0.000 | 0.000 | 0.000 | 0.000 | 0.000 | 0.000 | 0.000 | 0.000 | 0.000 | 0.000 | 0.000 |
| 18:1n9c | 53.162 | 90.235 | 31.273 | 133.188 | 122.137 | 60.998 | 35.675 | 28.181 | 29.515 | 33.509 | 30.394 | 32.593 | 111.551 | 35.967 | 0.000 |
| 18:2n6t | 0.000 | 4.113 | 1.209 | 2.695 | 2.439 | 1.265 | 0.000 | 0.000 | 0.628 | 1.091 | 1.397 | 0.000 | 2.036 | 0.918 | 5.640 |
| 18:2n6c | 8.916 | 11.753 | 10.173 | 14.948 | 19.529 | 6.366 | 14.096 | 14.096 | 9.364 | 6.006 | 5.874 | 7.021 | 19.412 | 8.708 | 25.703 |
| 18:3n6 | 1.277 | 0.000 | 0.000 | 1.302 | 1.127 | 0.000 | 0.000 | 0.000 | 1.054 | 0.000 | 1.721 | 2.821 | 0.000 | 0.950 | 0.000 |
| 18:3n3 | 6.589 | 28.770 | 22.449 | 49.788 | 61.808 | 12.234 | 6.285 | 6.285 | 27.969 | 14.260 | 3.053 | 3.897 | 58.666 | 4.978 | 87.841 |
| 20:00 | 0.000 | 1.148 | 4.306 | 1.607 | 0.000 | 1.056 | 3.994 | 3.994 | 1.410 | 0.538 | 1.065 | 1.663 | 3.029 | 1.866 | 2.810 |
| 20:1n9c | 16.671 | 15.626 | 18.544 | 12.963 | 0.000 | 21.611 | 30.164 | 22.483 | 8.542 | 17.769 | 16.695 | 21.016 | 30.563 | 37.802 | 29.358 |
| 20:2n6c | 3.102 | 1.110 | 2.358 | 2.313 | 3.072 | 1.890 | 1.919 | 1.919 | 1.156 | 2.444 | 0.000 | 2.165 | 3.512 | 4.559 | 0.000 |
| 20:3n6 | 16.737 | 12.810 | 17.685 | 10.252 | 9.404 | 19.948 | 29.276 | 26.555 | 7.446 | 17.126 | 18.060 | 16.940 | 26.749 | 36.818 | 26.791 |
| 20:4n6c | 4.290 | 1.919 | 4.679 | 3.795 | 3.001 | 1.112 | 2.985 | 2.985 | 2.036 | 0.918 | 6.118 | 9.776 | 3.145 | 2.374 | 5.103 |
| 20:3n3 | 0.932 | 2.712 | 0.955 | -15.698 | 2.415 | 2.092 | 0.000 | 0.000 | 2.328 | 1.252 | 0.000 | 0.000 | 2.024 | 1.477 | 3.777 |
| 22:00 | 5.114 | 0.661 | 3.315 | 0.772 | 0.795 | 3.737 | 1.436 | 1.436 | 0.634 | 0.834 | 2.338 | 0.684 | 12.648 | 6.067 | 15.176 |
| 20:5n3c | 0.000 | 115.970 | 132.833 | 0.000 | 166.846 | 85.739 | 110.258 | 110.258 | 94.567 | 77.742 | 110.537 | 124.771 | 197.458 | 96.423 | 293.571 |
| 22:1n9c | 1.149 | 0.802 | 1.257 | 163.145 | 1.953 | 0.595 | 0.000 | 0.000 | 1.335 | 0.652 | 0.354 | 0.838 | 2.771 | 6.239 | 0.000 |
| 22:2n6c | 2.038 | 1.436 | 2.085 | 0.730 | 0.631 | 2.548 | 2.096 | 2.096 | 0.566 | 1.143 | 1.318 | 1.047 | 1.476 | 2.797 | 0.000 |
| 23:00 | 14.287 | 12.657 | 17.629 | 8.119 | 7.526 | 17.876 | 23.948 | 19.274 | 6.068 | 13.667 | 14.519 | 0.671 | 1.400 | 32.116 | 23.060 |
| 24:00:00 | 10.295 | 8.734 | 15.141 | 0.978 | 0.602 | 0.227 | 0.000 | 0.000 | 4.858 | 10.418 | 11.304 | 2.250 | 13.994 | 24.321 | 15.880 |
| 24:1n9c | 7.636 | 10.711 | 4.319 | 1.200 | 18.821 | 0.143 | 0.874 | 0.874 | 0.611 | 6.567 | 5.067 | 4.523 | 0.000 | 5.847 | 22.501 |
| 22:6n3c | 63.877 | 91.872 | 68.368 | 97.146 | 124.917 | 78.665 | 56.519 | 56.519 | 74.501 | 61.343 | 40.713 | 37.100 | 17.035 | 36.184 | 151.263 |
